# Diagnostic efficiency of hybrid imaging using PSMA ligands, PET/CT, PET/MRI and MRI in identifying malignant prostate lesions

**DOI:** 10.1007/s12149-021-01606-7

**Published:** 2021-03-19

**Authors:** Sergiu Scobioala, Christopher Kittel, Heidi Wolters, Sebastian Huss, Khaled Elsayad, Robert Seifert, Lars Stegger, Matthias Weckesser, Uwe Haverkamp, Hans Theodor Eich, Kambiz Rahbar

**Affiliations:** 1 Department of Radiation Oncology, University Hospital Muenster, Albert-Schweitzer-Campus 1, 48149 Muenster, Germany; 2 Department of Pathology, University Hospital of Muenster, Muenster, Germany; 3 Department of Nuclear Medicine, University Hospital of Muenster, Muenster, Germany; 4 West German Cancer Center, Muenster and Essen, Germany

**Keywords:** Prostate cancer, PSMA-PET, Multiparametric MRI, Hybrid imaging, Biopsy-derived landmarks

## Abstract

**Objective:**

The objective of this study was to assess the accuracy of ^68^Ga-PSMA-11 PET/MRI, ^18^F-PSMA-1007 PET/CT, ^68^Ga-PSMA-11 PET/CT, and multiparametric (mp)MRI for the delineating of dominant intraprostatic lesions (IPL).

**Materials and methods:**

35 patients with organ-confined prostate cancer who were assigned to definitive radiotherapy (RT) were divided into three groups based on imaging techniques: ^68^Ga-PSMA-PET/MRI (*n* = 9), ^18^F-PSMA-PET/CT (*n* = 16) and ^68^Ga-PSMA-PET/CT (*n* = 10). All patients without PSMA-PET/MRI received an additional mpMRI. PSMA-PET-based automatic isocontours and manual contours of the dominant IPLs were generated for each modality. The biopsy results were then used to validate whether any of the prostate biopsies were positive in the marked lesion using Dice similarity coefficient (DSC), Youden index (YI), sensitivity and specificity*.* Factors that can predict the accuracy of IPLs contouring were analysed.

**Results:**

Diagnostic performance was significantly superior both for manual and automatic IPLs contouring using ^68^Ga-PSMA-PET/MRI (DSC/YI SUV_70%_—0.62/0.51), ^18^F-PSMA-PET/CT (DSC/YI SUV_70%_—0.67/0.53) or ^68^Ga-PSMA-PET/CT (DSC/YI SUV_70%_—0.63/0.51) compared to mpMRI (DSC/YI—0.47/0.41; *p* < 0.001). The accuracy for delineating IPLs was not improved by combination of PET/CT and mpMRI images compared to PET/CT alone. Significantly superior diagnostic accuracy was found for large prostate lesions (at least 15% from the prostate volume) and higher Gleason score (at least 7b) comparing to smaller lesions with lower GS.

**Conclusion:**

IPL localization was significantly improved when using PSMA-imaging procedures compared to mpMRI. No significant difference for delineating IPLs was found between hybrid method PSMA-PET/MRI and PSMA-PET/CT. PSMA-based imaging technique should be considered for the diagnostics of IPLs and focal treatment modality.

**Supplementary Information:**

The online version contains supplementary material available at 10.1007/s12149-021-01606-7.

## Introduction

Accurate delineation of the malignant intraprostatic lesions (IPLs) can be performed using advanced molecular imaging procedure as positron emission tomography (PET) technology and multiparametric (mp) MRI. Previous studies showed superior accuracy of [^11^C]-choline-PET/CT images compared to MRI for delineating IPLs [1–4]. Diagnostic performance of PET-CT methods for delineating IPLs was considerably improved with introduction of PSMA ligands such as ^68^Ga-PSMA and ^18^F-PSMA [5–10]. Recent studies evaluated the hybrid imaging tool ^68^Ga-PSMA-PET/MRI and ^18^F-PSMA-PET/MRI for the diagnostics of primary or recurrent prostate cancer (PCa) [6, 11–13]. It is expected that PSMA-PET/MRI should deliver higher diagnostic accuracy because it combines molecular, functional and morphological information [10]. As reported by Kim et al., the ^18^F-PSMA-PET/MRI provided higher detection rate of PCa than other imaging procedures [13]. Eiber et al. received superior accuracy for identifying the IPLs using ^68^Ga-PSMA-PET/MRI compared to stand alone ^68^Ga-PSMA-PET and mpMRI [6]. To date, the accuracy of PET or MRI imaging for delineating IPLs was evaluated using prostatectomy specimens as standard of reference [1, 2, 4, 6–8, 14]. Alternatively, for the purposes of radiotherapy (RT) planning with focal dose escalation on tumour areas within the prostate, the methodologies utilizing the biopsy-derived prostate specimens should be evaluated. In addition, RT concepts for primary PCa using image-guided focal boost treatment need an optimal diagnostic algorithm for localizing malignant IPLs. The present study evaluates diagnostic performance for identifying IPLs analysing the ^68^Ga-PSMA-PET/MRI, as well as ^18^F-PSMA-PET/CT and ^68^Ga-PSMA-PET/CT alone or in combination with mpMRI using the prostate biopsy specimens for the validation of diagnostic accuracy of images. In addition, parameters that can potentially predict the accuracy of PSMA-PET and MRI images for delineating IPLs were analysed.

## Materials and methods

### Patients and study strategy

This study is based on the analysis of 35 patients with low-to-high risk prostate carcinoma who were treated by external beam RT between years 2013 and 2019 in our department. The median age was 68 years (range 58–77 years) and the median pre-imaging PSA was 15.4 ng/mL (range 0.6–57.9 ng/mL). The patient, prostate carcinoma and imaging characteristics are presented in detail in Table 1. The patients were divided into three groups in dependence of performed imaging procedures: ^68^Ga-PSMA-PET/MRI (*n* = 9), ^18^F-PSMA-PET/CT (*n* = 16) and ^68^Ga-PSMA-PET/CT (*n* = 10). Additional mpMRI was performed in patients that received PET/CT screening. Only patients who had at least one tumour lesion greater than or equal to 1 cm^3^ and a SUV threshold of 3 were included in the study. Patients who had significant imaging artefacts and poor quality of biopsy specimens were excluded from the analysis.

**Table 1:** Characteristics of variables: patients, tumour, radiological method

Parameter	*n* (%), or range
No. of patients	35 (100)
Median age/range, years	68/58–77
Median prostate volume/range, cc	31/21–54
Pre-imaging PSA median/range, ng/mL	15.4/0.6–57.9
T stage	
cT1c	15 (43)
cT2a	7 (20)
cT2b	8 (23)
cT2c	5 (14)
Prostate biopsy Gleason score	
6	8 (23)
7a	6 (17)
7b	5 (14)
8	10 (28)
9	6 (17)
Radiological method	
^68^Ga-PSMA-PET/CT + mpMRI	10 (28)
^18^F-PSMA-PET/CT + mpMRI	16 (46)
^68^Ga-PSMA-PET /MRI	9 (26)
mpMRI	26 (74)
SUV values	
SUV_max_^68^Ga-PSMA-PET/CT	8.2 (2.6–19.2)
SUV_max_^18^F-PSMA-PET/CT	9.1 (2.1–21.4)
SUV_max_^68^Ga-PSMA-PET/MRI	7.8 (1.9–17.5)
SUV_70%_^68^Ga-PSMA-PET/CT	15 (23)^b^
SUV_70%_^18^F-PSMA-PET/CT	19 (27)^b^
SUV_70%_^68^Ga-PSMA-PET/MRI	14 (22)^b^
Intraprostatic tumor volume (%)^a^	
^68^Ga-PSMA-PET/CT	18.3/6.8–70.4
^18^F-PSMA-PET/CT	21.2/6.7–72.9
^68^Ga-PSMA-PET /MRI	19.7/7.2–74.6
T2W-MRI	17.8/6.4–71.4
DFW-MRI	18.1/6.3–69.4
DCE-MRI	18.7/6.3–68.5
Number of selected prostate lesions^c^	
^68^Ga-PSMA-PET/CT + mpMRI	21
^18^F-PSMA-PET/CT + mpMRI	33
^68^Ga-PSMA-PET/MRI	17

*PSA* prostate-specific antigen, *SUV*_*max*_ maximum standardized uptake value, *SUV*_*70%*_ 70% of the maximum SUV (defined as the best automatic contouring method), ^68^Ga*-/*^*18*^*F-PSMA* ^68^Gallium-/^18^Fluoromethylcholine-prostate specific membrane antigen, *PET* positron emission tomography, *mpMRI* multiparametric magnetic resonance image, *T2W* T2-weighted, *DFW* diffusion-weighted, *DCE* dynamic contrast-enhanced

^a^Intraprostatic tumor volume relative to total prostate volume

^b^Percentage of SUV_70%_ relative to all identified SUV-positive lesions

^c^Prostate lesions selected for the correlation analysis with a size of at least 1 cm^3^ and a SUV threshold of 3

All patients were reviewed at our institutional interdisciplinary tumor board. Informed consent was given.

### Image acquisition

^68^Ga-PSMA-11 and ^18^F-PSMA-1007 precursors were obtained from ABX (Advanced Biochemical Compound, Radeberg, Germany) and produced according to standard operation procedure described before [15–19]. The main advantage of ^18^F-PSMA-1007 is due to the higher amount of activity of cyclotron-produced ^18^F compared to ^68^Ga-PSMA-11 derived from ^68^Ge/^68^Ga generator elution and its higher half-life and higher physical spatial resolution [20]. In addition, ^18^F-PSMA-1007 has a very low urinary elimination, which is another advantage making it easier to differentiate between local recurrence or lymph node metastases and urinary activity in the ureter or the urinary bladder [15, 21]. Scanning for ^18^F-PSMA-1007 was performed 120 min and for ^68^Ga-PSMA-11 60 min p.i. starting at lower limbs to the scull. Patients were asked to empty the bladder before the scan. Images were acquired with a scan time of 3 min per bed position on a Siemens mCT scanner or Siemens PET-MRI (Siemens Healthcare, Knoxville, Tennessee, USA). Image reconstruction was performed using standard manufacturer software. For attenuation correction, a low-dose CT or mRAC was performed in parallel to PET images.

Prebiopsy mpMRI (*n* = 26) was performed on a 3 T system (Aero and Avanto, Siemens; Germany). The following MRI sequences were analyzed: T2-weighted (T2W) imaging, diffusion-weighted imaging (DFW), apparent diffusion coefficient map (ADC-map), and dynamic contrast-enhanced (DCE) perfusion imaging. Maps of ADC are computed from DWI and provide a quantitative parameter to evaluate prostate regions with suspicion of prostate carcinoma. Multi-vendor digital imaging system “DynaCAD” was used for performing real-time image analysis of prostate MRI (“Sanova Medical Systems”, Austria, Vienna). The receiver coil technology included pelvic phased-array coils without the addition of an endorectal coil. The median *b* value was 1.700 (range 900–2000). Image data were analyzed using the PIRADS v2.1 classification [22]. Intraprostatic lesions (IPLs) rated as score 4 or greater were selected for a targeted biopsy. Detailed technical description of the mpMRI acquisition protocol is given in [23].

### Prostate punch biopsy

In accordance with current guidelines for prostate carcinoma, at least 10–12 puncture specimens were obtained [24, 25]. Most of the analyzed patients (70%) underwent systematic and MRI-targeted biopsies [26–28]. Following prostate biopsy, formalin-fixed, paraffin-embedded tissue samples were cut into 4-μm-thick sections, prepared on slides, and stained with hematoxylin and eosin. Gleason-score and tumor extent were reported for each localization following the clinical practice guidelines [29, 30].

### Definition of IPL contours

**Fig. 1:**
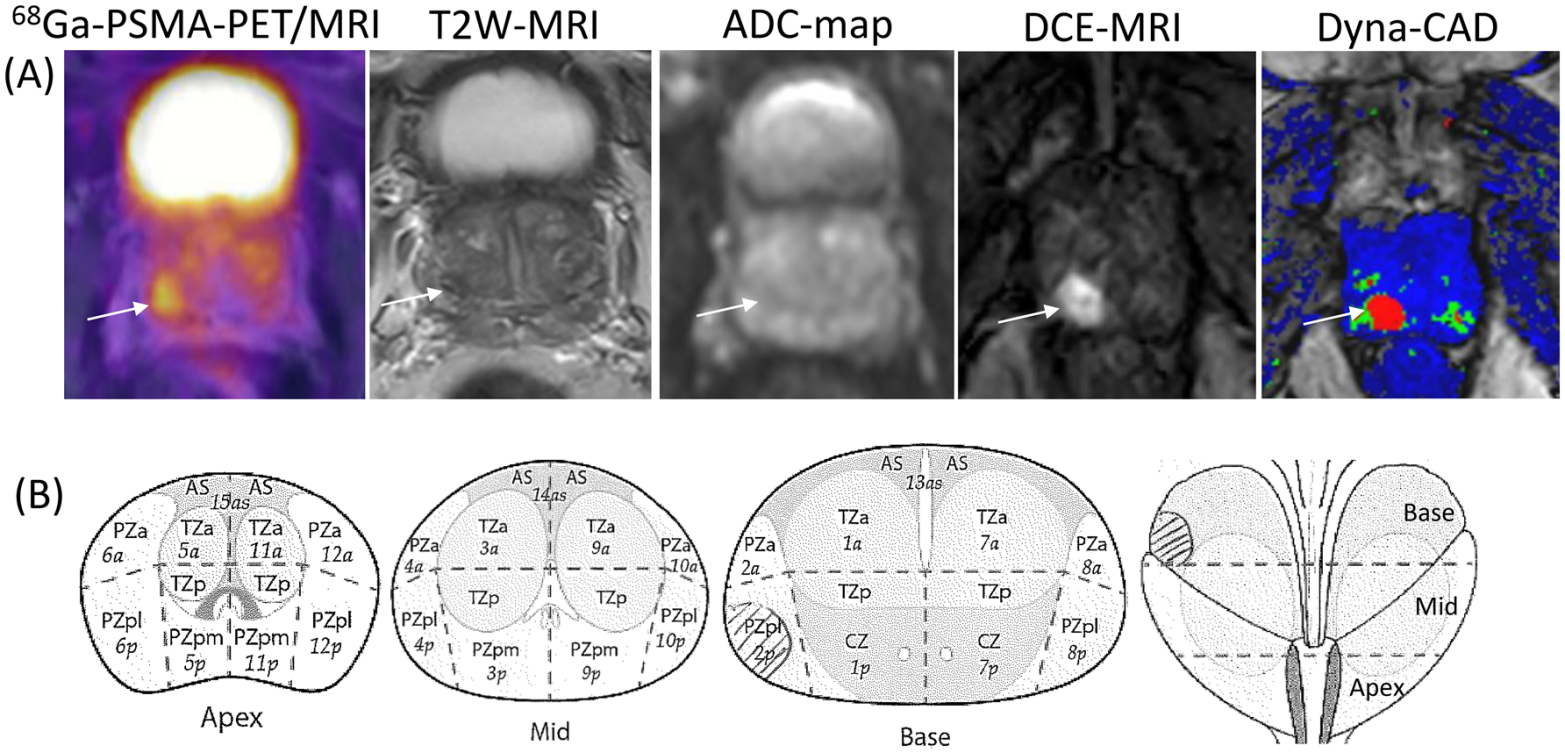
Transverse slices showing the IPL contours in peripheral zone of the base region in right lobe using the hybrid method ^68^Ga-PSMA-PET/MRI and the PIRADS-Map in patient with a low-risk prostate carcinoma (iPSA 9.2 ng/mL; Gleason score 6). **a** The lesion (arrow) shows intense focal uptake of radioligand ^68^Ga in PSMA-PET (SUVmax 13.0), hypointense signal in T2W image, restricted diffusion in low-signal mass on ADC-map, and marked enhancement on DCE image. The lesion is displayed in color by the use of multi-vendor digital imaging system “DynaCAD”; **b** schematic illustration of the tumor regions (crossed out) in biopsy specimen axial and coronal slices designated in peripheral zone of the base region of the dorsal right lobe. *PSMA* prostate-specific membrane antigen, *PET* positron emission tomography, *MRI* magnetic resonance imaging, *T2W* T2-weighted imaging, *ADC-map* apparent diffusion coefficient map, *DCE* dynamic contrast-enhanced

Delineation of IPLs was performed separately in PSMA-PET/MRI, PSMA-PET/CT and mpMRI images. The IPLs found in the imaging modalities were marked in the PIRADS schemes [22]. It was then checked whether prostate carcinoma had also been found in these regions in the diagnostic biopsies (Fig. 1, “Supplemental data”).

Manual registration of the PIRADS-Map and the PET/CT or MRI was performed with different software tools. At the beginning, the landmarks were sliced in the axial images and divided into 12 segments at each of the apex, mid and base prostate region. The axial slice of PET/CT (5-mm slice thickness) or MRI (3-mm slice thickness) visualizing the apex, mid or base prostate region of the PIRAD schemes were selected and exported to GIMP 2.10.14 (free GNU Image Manipulation Program). A difference in image size was corrected and the slices were registered. The first step of registration is a rigid registration. Only translation and rotational manipulation is, therefore, allowed. This registered image pair was then exported to Adobe Illustrator CS5 (Adobe Systems Incorporated; San Jose, CA, USA). Anatomical differences between the patient PET/CT or MRI and the standard PIRAD-Map were corrected in a second step using multipoint deformation. For this the contour of the apex, mid or base prostate region of the PIRAD-Maps were selected and multi-deformation-points at the contour were added. By selecting the deformation points at the contour, the whole PIRAD-Map was deformed. This deformation was finished when the PIRAD-Map and the anatomy of the prostate are as close as possible. Two experienced radiation oncologists performed the manual deformation by the matching of PET/CT or MRI images with PIRADS-Map independently. The radiation oncologist’s matchings were verified in blinded conditions by another radiation oncologist. The registration process was performed in two-dimensional accuracy at different axial slices (apex, mid or base prostate region).

Two experienced radiation oncologists and two nuclear medicine physicians were independently involved in the manual contouring of intraprostatic foci on the co-registered PET and MRI scans. We also performed interobserver analyses to account for discrepancies in manual delineation of IPLs. Figure 1 shows the identifying of malignant prostate lesion using the hybrid method ^68^Ga-PSMA-PET/MRI with separate delineation of prostate focus in PSMA-PET image and different MRI sequences. In addition, the IPLs were identified using the PSMA-PET-based automatic contours, applying the threshold’s set of maximal standardized uptake values (SUV_max_) within the prostate from 40 to 80% [4]. An isotropic line of 5 mm was added to the external margin both of image-defined IPLs and of biopsy contour [22, 31].

For correlation analyses, we selected the dominant IPLs based on the criteria that regard the size and SUV activity of identified intraprostatic foci [32–35]. IPLs with a size of at least 1 cm^3^ and a SUV threshold of at least three were defined as dominant IPLs considering that smaller carcinoma foci expressing low SUV activity may be poorly detected and characterized by PET/CT or MRI techniques.

### Correlation analysis

Dice similarity coefficient (DSC), Youden index (YI), sensitivity and specificity were evaluated on the basis of matching between radiological images and biopsy landmarks [14, 36–38]. Detailed description of the correlation parameters is presented in the “Supplemental Data”.

The contouring accuracy was separately evaluated for all images, and received values were averaged and designated as ^68^Ga-PSMA-PET/MRI_man_, ^18^F-PSMA-PET/CT_man_, ^68^Ga-PSMA-PET/CT_man,_ and mpMRI_man._ The contouring method showing the highest DSC and YI values was determined as the best automatic contouring method [4].

The following factors that may be predictive for the contouring accuracy were analysed: pre-imaging PSA, prostate biopsy Gleason score (GS), relative IPL volume (percentage of the tumour volume to the total prostate volume), IPL localisation, SUV_max_. For this purpose, the effect of these factors on the DSC results was evaluated [4].

### Statistical analysis

The Kolmogorov–Smirnov test and the Kruskal–Wallis tests were used to evaluate abnormally distributed data and to compare the correlation parameters, including DSC, YI, sensitivity and specificity, between PSMA-PET/CT and MRI images. The influence of the following indicators as initial PSA, biopsy GS, tumor volume, tumor localisation, and SUV_max_ on the accuracy of PSMA-PET/CT and mpMRI for identifying the IPLs was analyzed using the Pearson’s Correlation Coefficient. A multivariate logistic regression analysis was performed. The threshold for statistical significance was *p* < 0.05. As the nature of the study was exploratory, *p* values were interpreted from a Bayesian point of view. Statistical analyses were performed using SPSS software (IBM SPSS Statistics 24.0).

## Results

Patient and radiological image characteristics are presented in Table 1. Principal agreement for delineating IPLs was observed between the imaging techniques. Localisations of positive biopsies exceeded the IPLs in the majority of patients. Based on the criteria for dominant IPLs, we selected for the correlation analysis 17 prostate lesions identified with ^68^Ga-PSMA-PET/MRI, 33 IPLs detected with ^18^F-PSMA-PET/CT and mpMRI, and 21 IPLs diagnosed with ^68^Ga-PSMA-PET/CT and mpMRI (Table 1). Interobserver analysis revealed no relevant variabilities in manual contouring of IPLs.

**Fig. 2:**
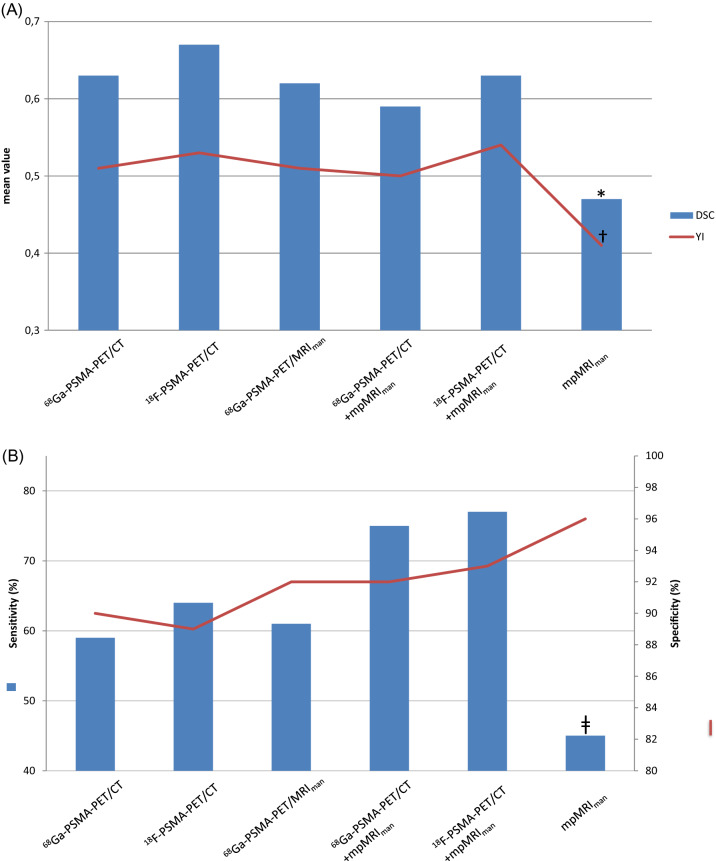
**a** Mean dice similarity coefficients (DSC) (blue bars) and the mean Youden index (red line) of different radiological methods; **b** comparison of sensitivity (blue bars) and specificity (red line) of the different radiological methods. The presented mean values were assessed using the best automatic contouring method (defined for SUV_70%_) as well as on the basis of manual (man) contouring. The correlation indices, including the mean DSC (*), YI (^†^), and sensitivity (^ǂ^) were found significantly lower (*p* < 0.001) for mpMRI compared to ^68^Ga-PSMA-PET/CT and ^18^F-PSMA-PET/CT alone or in combination with mpMRI, as well as to ^68^Ga-PSMA-PET/MRI. *PSMA* prostate-specific membrane antigen, *PET* positron emission tomography, *MRI* magnetic resonance imaging

The correlation indices, including the mean DSC, YI, sensitivity and specificity for each contouring value are summarized in Table 2. SUV_70%_ was defined as the best automatic contouring method showing the highest DSC and YI values both for ^68^Ga-PSMA-PET/CT and ^18^F-PSMA-PET/CT. The DSC values for ^68^Ga-PSMA-PET/CT and ^18^F-PSMA-PET/CT were found significantly superior compared to mpMRI (*p* < 0.001). The differences in DSC values between PSMA-PET images, including ^68^Ga-PSMA-PET/MRI, were not significant. No improvements in DSC values were found by additional use of mpMRI to PSMA-PET techniques (Fig. 2a, Table 2).

**Table 2:** Correlation indices evaluated by the co-registration of PSMA-PET and MRI images with biopsy specimens

Parameter	DSC	YI	Sensitivity (%)	Specificity (%)
SUV_3.0_^68^Ga-PSMA-PET/CT	0.42 ± 0.17	0.33 ± 0.14	57 ± 9	88 ± 18
SUV_3.0_^18^F-PSMA-PET/CT	0.45 ± 0.10	0.38 ± 0.12	61 ± 8	90 ± 18
SUV_40%_^68^Ga-PSMA-PET/CT	0.51 ± 0.15	0.40 ± 0.13	95 ± 21	41 ± 14
SUV_50%_^68^Ga-PSMA-PET/CT	0.54 ± 0.11	0.44 ± 0.13	93 ± 23	59 ± 19
SUV_60%_^68^Ga-PSMA-PET/CT	0.58 ± 0.15	0.47 ± 0.16	83 ± 20	76 ± 21
SUV_70%_^68^Ga-PSMA-PET/CT	0.63 ± 0.24	0.51 ± 0.22	59 ± 17	90 ± 18
SUV_80%_^68^Ga-PSMA-PET/CT	0.49 ± 0.13	0.38 ± 0.19	45 ± 23	93 ± 12
SUV_40%_^18^F-PSMA-PET/CT	0.55 ± 0.21	0.44 ± 0.18	97 ± 25	42 ± 18
SUV_50%_^18^F-PSMA-PET/CT	0.57 ± 0.23	0.45 ± 0.17	94 ± 21	62 ± 20
SUV_60%_^18^F-PSMA-PET/CT	0.62 ± 0.20	0.49 ± 0.21	86 ± 19	79 ± 24
SUV_70%_^18^F-PSMA-PET/CT	0.67 ± 0.18	0.53 ± 0.20	64 ± 23	89 ± 15
SUV_80%_^18^F-PSMA-PET/CT	0.53 ± 0.16	0.41 ± 0.24	54 ± 20	93 ± 15
^68^Ga-PSMA-PET/MRI_man_	0.61 ± 0.21	0.50 ± 0.18	79 ± 17	97 ± 17
SUV_70%_^68^Ga-PSMA-PET/MRI	0.62 ± 0.14	0.51 ± 0.37	61 ± 13	96 ± 11
^68^Ga-PSMA-PET/CT + mpMRI_man_	0.59 ± 0.24	0.50 ± 0.23	75 ± 19	92 ± 21
^18^F-PSMA-PET/CT + mpMRI_man_	0.63 ± 0.19	0.54 ± 0.23	77 ± 23	93 ± 16
mpMRI_man_	0.47 ± 0.13^a^	0.41 ± 0.16^b^	45 ± 9^c^	96 ± 9

Values are given with ± standard deviation

*DSC* Dice similarity coefficient, *mpMRI*_*man*_ multiparametric magnetic resonance imaging (MRI) manual contour, ^68^Ga*-/*^*18*^*F-PSMA* ^68^Gallium-/^18^Fluoromethylcholine-prostate specific membrane antigen, *PET*_*man*_ positron emission tomography/manual contouring, *SUV* standard uptake value, *YI* Youden index

^a^DSC mpMRI_man_ *vs.* ^68^Ga-PSMA-PET/MRI_man,_^68^Ga-PSMA-PET/CT + mpMRI_man,_ and ^18^F-PSMA-PET/CT + mpMRI_man_ (*p* < 0.001)

^b^YI mpMRI_man_ vs*.* ^68^Ga-PSMA-PET/MRI_man,_^68^Ga-PSMA-PET/CT + mpMRI_man,_ and ^18^F-PSMA-PET/CT + mpMRI_man_ (*p* < 0.001)

^c^Sensitivity mpMRI_man_ vs*.* ^68^Ga-PSMA-PET/MRI_man_, ^68^Ga-PSMA-PET/CT + mpMRI_man_, and ^18^F-PSMA-PET/CT + mpMRI_man_ (*p* < 0.001)

In accordance to DSC, the median YIs were found significantly superior for ^68^Ga-PSMA-PET/CT and ^18^F-PSMA-PET/CT compared to mpMRI. The median YIs were not improved by additional use of mpMRI (Fig. 2a, Table 2). The sensitivity and specificity, as expected, showed the correlation with YI (Table 2). A trend to the higher specificity was found for ^68^Ga-PSMA-PET/MRI (97%, *p* = 0.19) compared to ^18^F-PSMA-PET/CT (93%) or ^68^Ga-PSMA-PET/CT (92%) (Table 2)*.* The mpMRI revealed the lower sensitivity (45%, *p* < 0.001) and a tendency to higher specificity (96%, *p* = 0.24) compared to ^18^F-PSMA-PET/CT or ^68^Ga-PSMA-PET/CT techniques (Fig. 2b).

As demonstrated in Table 3, IPL localisation and relative IPL volume were found as factors significantly predicting the accuracy of PSMA-PET/CT and mpMRI for localizing the IPLs. Superior diagnostic accuracy was found for lesions with a minimal relative volume of 15%. ^18^F-PSMA-PET/CT showed higher diagnostic performance than ^68^Ga-PSMA-PET/CT for lesions localized in transition zone of the base region (0.67 vs. 0.56, correspondingly, *p* = 0.003). The difference in correlation results between ^68^Ga-PSMA-PET/CT and ^18^F-PSMA-PET/CT in mid and apex regions was not significant. Diagnostic accuracy of IPLs with GS 7b was superior to GS 6 and 7a (*p* < 0.05). The pre-imaging PSA and SUV_max_ showed no effect on diagnostic performance of PSMA-PET/CT tools.

**Table 3:** Factors predicting the accuracy of PSMA-PET techniques and mpMRI for delineating IPLs

Parameter	^18^F-PSMA-PET/CT		^68^Ga-PSMA-PET/CT		^68^Ga-PSMA-PET/MRI		mpMRI	
	*r*	*p*	*r*	*p*	*r*	*p*	*r*	*p*
IPL localisation								
Apex	0.18	0.42	0.15	0.29	0.24	0.37	0.12	0.53
Mid	0.35	0.24	0.12	0.47	0.19	0.45	0.17	0.39
Base	0.92	0.03^a^	0.43	0.21	0.46	0.24	0.14	0.42
IPL volume^b^								
≤ 5%	0.22	0.36	0.27	0.41	0.25	0.28	0.19	0.41
≤ 10%	0.64	0.13	0.68	0.09	0.71	0.11	0.52	0.22
≤ 15%	0.92	0.02^a^	0.93	0.02^a^	0.94	0.03^a^	0.67	0.09
≤ 20%	0.96	0.01^a^	0.96	< 0.01^a^	0.97	< 0.01^a^	0.84	0.04^a^
Gleason score								
6	0.18	0.45	0.24	0.36	0.26	0.30	0.13	0.41
7a	0.38	0.20	0.43	0.17	0.53	0.09	0.31	0.27
7b	0.79	0.051	0.81	0.04^a^	0.84	0.03^a^	0.56	0.17
8	0.87	0.04^a^	0.90	0.03^a^	0.92	0.02^a^	0.81	0.07
Pre-imaging PSA (ng/mL)								
≤ 10	0.27	0.32	0.18	0.35	0.34	0.23	0.21	0.24
10–20	0.30	0.37	0.33	0.26	0.28	0.52	0.19	0.41
> 20	0.37	0.29	0.25	0.41	0.46	0.17	0.27	0.37
SUV_max_								
2	0.31	0.23	0.25	0.35	0.27	0.26	–	–
3	0.28	0.40	0.23	0.28	0.36	0.21	–	–
≥ 4	0.44	0.15	0.37	0.20	0.33	0.30	–	–

^68^Ga*-/*^*18*^*F-PSMA* ^68^Gallium-/^18^Fluoromethylcholine-prostate specific membrane antigen, *PET* positron emission tomography, *mpMRI* multi-parametric magnetic resonance imaging, *IPL* intraprostatic lesion, *r* Pearson’s Correlation Coefficient, *PSA* prostate specific antigen, *SUV*_*max*_ maximal standardized uptake value

^a^5% level of significance

^b^Relative to the whole prostate volume

## Discussion

This study evaluates the diagnostic accuracy of PSMA-PET-based technologies as well as mpMRI for localizing IPLs using prostate biopsy specimens as standard of reference. Previous studies evaluated the diagnostic performance of PET/CT methods and mpMRI for delineating IPS using the prostatectomy patterns [5–9, 11, 13].

Eiber and colleagues revealed significant superiority for ^68^Ga-PSMA-PET/MRI compared to separate PET/CT and mpMRI techniques for delineating malignant intraprostatic foci [6]. Our results show no relevant difference in accuracy for delineating IPLs between ^68^Ga-PSMA-PET/CT and ^18^F-PSMA-PET/CT and ^68^Ga-PSMA-PET/MRI. However, a trend to superior specificity was seen using the hybrid method. This discrepancy can be explained given different radiological and morphological criteria for the definition of dominant or malignant IPLs between the studies. In detail, Eiber et al. differentiated the malignancy of IPLs in PET and MRI images using 5-point Likert scale [6]. Based on the criteria for superior malignancy of intraprostatic foci, we selected dominant lesions with a SUV threshold of at least 3 and size of at least 1 cm^3^ considering that smaller carcinoma foci expressing low SUV activity do not determine the risk of local relapse after prostatectomy and may be poorly detected and described by PET/CT or MRI techniques [14, 32–35]. Second, manual contouring of IPLs as a subjective method can also provoke the deviations in correlation results across the studies. To reduce the inaccuracy trough the manual contouring, we generated the automatic contours based on the thresholds set of the SUVmax values inside the prostate as described by Chang et al. [4]. In addition, the correlation analyses can be affected through inaccuracy in co-registration proceeding between images and histological landmarks. Advanced software providing the registration with minimal deformation of image scans and landmarks should be analysed in further studies. Despite some diagnostic limitations, the mpMRI was recommended in several guidelines as staging method as well as method for targeted biopsy in the diagnostic of primary PCa [26–28, 39–44]. In agreement with other studies, both manual and automatic contouring using PSMA-PET techniques showed more accuracy compared to mpMRI for localizing IPLs when correlated with reference biopsy results [4–8]. With the exception of superior specificity, no improvement in correlation outcome was received by additional use of mpMRI. mpMRI was used as an additional diagnostic method to identify the IPLs. Separately, MRI was applied for prostate punch biopsy. Superior correlation between histological results and diagnostic mpMRI can be expected for patients with the MRI-derived prostate biopsies.

As demonstrated in previous studies, the rate of not detected intraprostatic foci by separate use of MRI-targeted biopsy and systematic biopsy remains relatively high (up to 20%) [26–28]. Superior detection rate can be achieved by combined use of systematic biopsy and MRI-targeted biopsy [26–28]. For this reason, we used a combination of both biopsy modes in the most of analysed patients (70%) reducing the risk of not detected prostate carcinoma foci. In accordance to several previous studies, we performed the correlation analysis using the “section model” [1–3, 6–8]. Alternatively, a “voxel model” was recommended by another group of authors [4, 14, 31]. Using the much more voxels per prostate volume, superior delineation of IPLs can be potentially achieved. However, the section model seems to be more compatible to the two-dimensional registration between images and histological specimens performed in our study. And the studies where the voxel model was used, a three-dimensional registration were performed. Prostatectomy specimens may serve as a more conclusive reference, as previous studies localized IPLs on 3–5 mm transverse slice of the prostate [4, 7, 8, 14]. This provided a better diagnostic performance compared to biopsy-based investigations such as the present study where the landmarks were sliced in the axial images and divided into 12 segments at each of the apex, mid and base prostate region. However, obtaining biopsy specimens is the most promising reference in evaluating IPLs in non-surgical radiotherapy treatment regimens given the absence of whole-organ prostatectomy specimens.To our opinion, the combination of histological landmarks generated on the basis of MRI-targeted and systemic biopsies with advanced PSMA-PET/MRI or PET/CT technologies can be recommended for localizing malignant prostate foci.

The IPL localization and volume, as well as GS were found as factors predicting the accuracy of PSMA-PET/CT and mpMRI for delineating IPLs. Strong positive correlation with DSC was observed for prostate foci localized in the base region and not in mid or apex regions. Thus, significantly higher correlation was found for ^18^F-PSMA-PET/CT versus ^68^Ga-PSMA-PET/CT localizing the IPLs in transition zone of the base region. This phenomenon can be explained by lack of physiological SUV-activity in urinary bladder using the radioligand ^18^F-PSMA in contrast to ^68^Ga-PSMA. Large lesions and high GS showed superior correlation outcome both for PSMA-PET/CT and mpMRI techniques. Thus, the PET/CT and MRI methods showed superior diagnostic accuracy of prostate lesions with at least 15% of prostate volume and GS 7b and higher. Similarly, Eiber and colleagues showed superior diagnostic score for prostate foci > 5 mm and GS ≥ 7 using the hybrid method ^68^Ga-PSMA-PET/MRI [6]. As known from the literature, diagnostic performance of primary or recurrent prostate cancer by PET or MRI techniques is in positive correlation with absolute PSA values [5, 10, 45]. In contrast, we showed no positive correlation between PSA values and diagnostic performance of PSMA-PET/CT or mpMRI. This result can be related with discrepancy between PSA level and grade of GS in about 20% of cases. These patients revealed the GS 7b or 8 by PSA values not exceeding 10 ng/mL. In the same manner, Eiber et al. showed no effect of PSA on diagnostic quality of PSMA-PET/CT for localizing the IPLs [6].

Several trials validated the efficiency of PET/CT-based approaches for RT planning using focal dose escalation on intraprostatic tumour areas [7, 8, 46–49]. The results reported here show that all analyzed PSMA-PET-based methods, inclusive the hybrid method ^68^Ga-PSMA-PET/MRI, seem to be accurate for delineating malignant IPLs. Importantly, this is the first study that uses biopsy specimens as the standard of reference for identifying IPLs. To facilitate this, we specifically designed biopsy-derived PIRAD-Maps that can be used as reference models for localizing the IPLs. The second crucial question concerns the selection criteria for dominant IPLs. It is known that both SUV activity and the size of intraprostatic foci determine the malignancy of IPLs. However, the threshold values for both of these parameters for selection of dominant IPLs are not precisely defined. An addition, it is not clear, if the focal boost treatment of the prostate lesions selected in PSMA-PET/CT would have a therapeutic advantage for reducing the risk of local relapse compared to the boost applying to the whole prostate. Particular relevance for focal dose escalation may have the prostate lesions showing the PSMA uptake after initiation of androgen deprivation therapy. In this perspective, optimisation in selection of dominant IPLs, biopsy guidelines, design of biopsy-derived landmarks, and technologies for registration of images and biopsy landmarks should be evaluated in further studies.

Summarizing the key findings of this study: (1) PSMA-PET/CT revealed superior diagnostic accuracy for delineating IPLs as compared to mpMRI; (2) no improvement in diagnostics of IPLs was shown by combined use of PSMA-PET/CT and mpMRI compared to PSMA-PET/CT alone; (3) no significant difference in diagnostic accuracy was observed between the hybrid method ^68^Ga-PSMA-PET/MRI and separate techniques ^18^F-PSMA-PET/CT and ^68^Ga-PSMA-PET/CT, however, the hybrid method showed a trend to a higher diagnostic specificity; (4) superior diagnostic performance for ^18^F-PSMA-PET/CT versus ^68^Ga-PSMA-PET/CT was found for lesions localized in the base region; and (5) the volume of lesions and the grade of GS showed a positive correlation with diagnostic accuracy of PSMA-PET/CT and mpMRI.

The limitations of this study are: (1) heterogeneous cohort of selected patients; (2) results can be affected by use of manual contouring of IPLs; and (3) it does suffer from inaccuracy during co-registration between imaging and biopsy landmarks.

## Conclusion

The PSMA-PET bears superior contouring of prostate lesions than mpMRI. No improvement for delineating IPLs was received by combination of PSMA-PET/CT and mpMRI compared to PSMA-PET/CT alone. Both PSMA-PET/CT and PSMA-PET/MRI techniques deliver high diagnostic effectivity for localizing the IPLs, without relevant difference between separate and hybrid methods. Positive correlation between diagnostic accuracy and tumor size as well as tumor GS was observed.

## Supplementary Information

Below is the link to the electronic supplementary material.

Supplementary file1 (DOCX 14 kb)

Supplementary file2 (PDF 414 kb)
