# Supplementary material for: Diagnostic efficiency of hybrid imaging using PSMA ligands, PET/CT, PET/MRI and MRI in identifying malignant prostate lesions
Source: Ann Nucl Med. 2021 Mar 19;35(5):628–38. doi: 10.1007/s12149-021-01606-7 (PMC8079339; doi:10.1007/s12149-021-01606-7)
Supplement: Supplementary file 1 — Supplementary file1 (DOCX 14 kb) [file 12149_2021_1606_MOESM1_ESM.docx]

**Correlation parameters**

Dice similarity coefficient (DSC) is described in detail in [1, 2]. The DSC was calculated as follows: *DSC=* *2 • A Ո B / (A + B)*, where ”A” is the volume of is the volume of tumor area outlines in biopsy landmark, “B” – is the volume of lesion defined in radiological imaging. Youden index (YI) – shows the overall correlation, and was calculated considering the sensitivity and specificity indices: *YI =* *Sensitivity + Specificity -1* [2]. Sensitivity – reflects the overlapping of prostate lesions defined in radiological images and biopsy specimens and calculated according to the following formula: *Sensitivity =* *TP / (TP + FN)*, where “TP” is a true positive, and “FN” is a false positive [2]. Specificity – reflects the lack of overlap between image- and biopsy-defined IPLs, and was calculated as: *Specificity =* *TN / (TN + FN)*, where “TN” is a true negative, and “FP” is a false positive [2].

**References:**

1. National Collaborating Centre for Cancer and National Institute for Health and Clinical Excellence (NICE), Prostate Cancer: diagnosis and treatment. 2008.

2. Chang JH, Joon DL, Lee ST, Gong SJ, Scott AM, Davis ID, Clouston D, Bolton D, Hamilton CS, Khoo V. Histopathological correlation of (11)C-choline PET scans for target volume definition in radical prostate radiotherapy. Radiother Oncol. 2011;99:187-192. https://doi:10.1016/j.radonc.2011.03.012
