# Supplementary material for: Diagnostic efficiency of hybrid imaging using PSMA ligands, PET/CT, PET/MRI and MRI in identifying malignant prostate lesions
Source: Ann Nucl Med. 2021 Mar 19;35(5):628–38. doi: 10.1007/s12149-021-01606-7 (PMC8079339; doi:10.1007/s12149-021-01606-7)
Supplement: Supplementary file 2 — Supplementary file2 (PDF 414 kb) [file 12149_2021_1606_MOESM2_ESM.pdf]

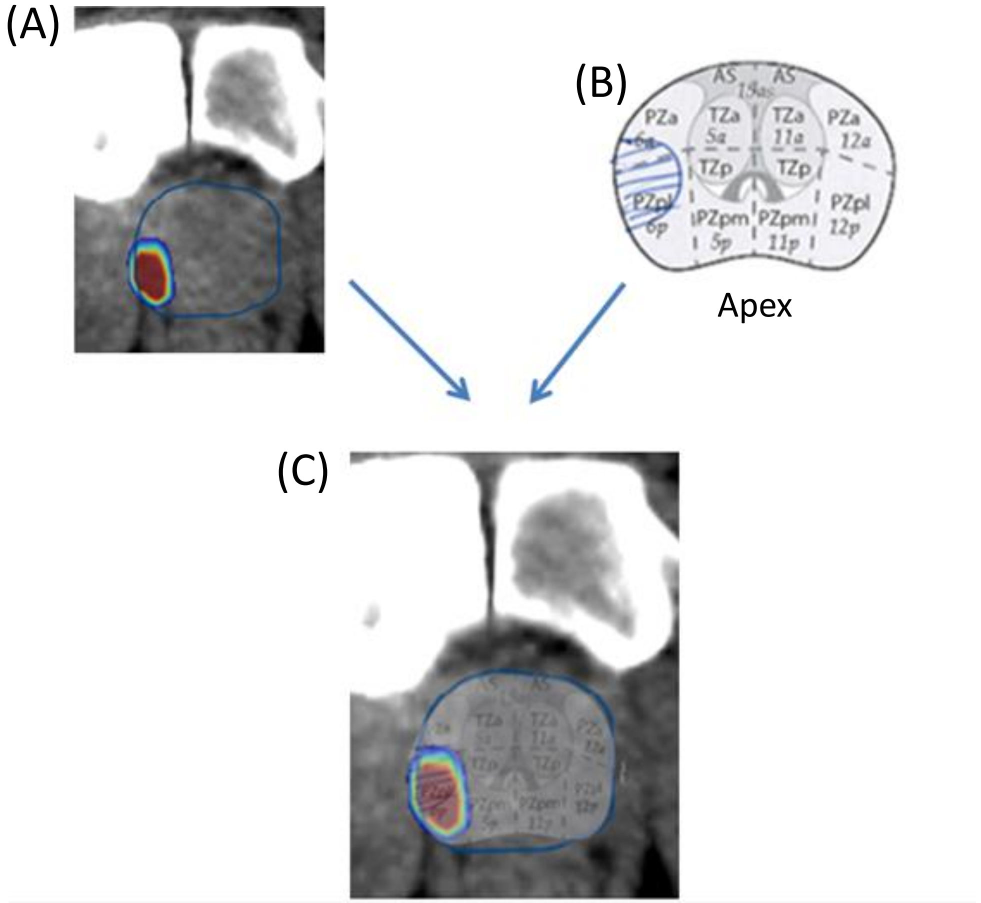

Figure 1. Image registration of ^{68}Ga-PSMA-PET/MRI and PIRADS-Map
The corresponding axial slice of the ^{68}Ga-PSMA-PET/MRI **(A)** and the PIRADS-Map **(B)** were matched in two steps. First, a rigid registration of ^{68}Ga-PSMA-PET/MRI and the PIRADS-Map was performed. Second, a multipoint deformation results in a matched image set **(C).**
